# Supplementary material for: Melatonin Maintains Postharvest Quality in Fresh Gastrodia elata Tuber by Regulating Antioxidant Ability and Phenylpropanoid and Energy Metabolism During Storage
Source: Int J Mol Sci. 2024 Nov 1;25(21):11752. doi: 10.3390/ijms252111752 (PMC11545936; doi:10.3390/ijms252111752)
Supplement: Supplementary file 1 [file ijms-25-11752-s001.zip › ijms-3271867-supplementary.pdf]

Supplementary Materials

Table S1. Primer sequences used for real-time PCR.

GENE	PRIMER SEQUENCE	GENBANK ID
GeSOD	Forward: GGTCTATGGCTTCGTCTCGG	GWHTBDNU007951
	Reverse: GTTCGCCCCCTTAGTGACAA	
GeCAT	Forward: GCCCTCAAACCGAACCCTAA	GWHTBDNU005072
	Reverse: GACACCGGAACCATCCATGT	
GeAPX	Forward: GCTGTGGATGAGGATGCCTT	GWHTBDNU008179
	Reverse: AGCAAACCCGAGCTCTGAAA	
GeH^{+}-ATPase	Forward: CGATGTCTCGAGGCAGATCC	GWHTBDNU027618
	Reverse: CGGAGATCTCGTTGGCCTTT	
GeCa^{2+}-ATPse	Forward: ATTCCCAGCTTTCGGACTGG	GWHTBDNU030933
	Reverse: TCATCGCCTTCACTTTCCCC	
GeSDH	Forward: CAAGGAGCCCATCCCTGTTT	GWHTBDNU000152
	Reverse: AGGCCCTGCCAAATACAACA	
GeCCO	Forward: GAGGACATCCAGCCGATAGC	GWHTBDNU026725
	Reverse: ATCCTTGGTCCCAAACGGAC	
GePAL	Forward: TTGCCCGGATTCATCGAGAG	GWHTBDNU000920
	Reverse: ATGGAGTGCCTTTCGTCTGG	
GeC4H	Forward: CTGAAGCTGAGGGTGCTCAA	GWHTBDNU016259
	Reverse: TGAGCCGAACTTCCTTCACC	
Ge4CL	Forward: TCCTGTCCAAATTCCAGGGC	GWHTBDNU001589
	Reverse: GACGTTTTCGCTTAGTCGCC	
Geactin	Forward: ATTCTTCCGCTCGATAGCCG	GWHGBDNU001679
	Reverse: GATGATGGGGATCGCAGGAG	
